# Treatment of hypertriglyceridemia and HIV: fenofibrate-induced changes in the expression of chemokine genes in circulating leukocytes

**DOI:** 10.1186/1742-6405-6-26

**Published:** 2009-11-23

**Authors:** Carlos Alonso-Villaverde, Gerard Aragonès, Raúl Beltrán-Debón, Laura Fernández-Sender, Anna Rull, Jordi Camps, Josep M Alegret, Jorge Joven

**Affiliations:** 1 Centre de Recerca Biomèdica, Hospital Universitari Sant Joan, IISPV, Universitat Rovira i Virgili, Sant Joan s/n, 43201 Reus, Spain

## Abstract

Fenofibrate changed the expression of chemokine genes in circulating leukocytes of HIV-infected patients with hypertriglyceridemia. The data suggest that fenofibrate when effective in the treatment of lipoprotein abnormalities, may act as a modulator of systemic inflammation. This particular action, therefore, may also influence the clinical course of the disease.

## Background

The impact of mutant chemokine ligands and receptors in the host susceptibility to HIV infection, in the clinical course of the disease and in the development of new therapies has been extensively described [1]. Chemokines have also been implicated in the higher rates of atherosclerosis observed in HIV-infected patients which has been partially attributed to either a direct effect of highly active antiretroviral therapies or the concomitant metabolic abnormalities [2-4]. Treatment of HIV infection, particularly with the use of protease inhibitors (PIs), can raise triglyceride levels to the threshold indicated for intervention. Therefore, the need to maintain viral suppression may be challenged by the need to treat abnormal lipid levels. Fibrates, ligands for peroxisome proliferator-activated receptor α (PPARα), represent an effective treatment for hypertriglyceridemia that reduce coronary events and delay progression of coronary atherosclerosis [5,6]. We hypothesized that such a treatment with fenofibrate may change the expression of chemokine genes in tissues and that this effect could be readily observed in circulating leukocytes.

## Methods

**Figure 1:**
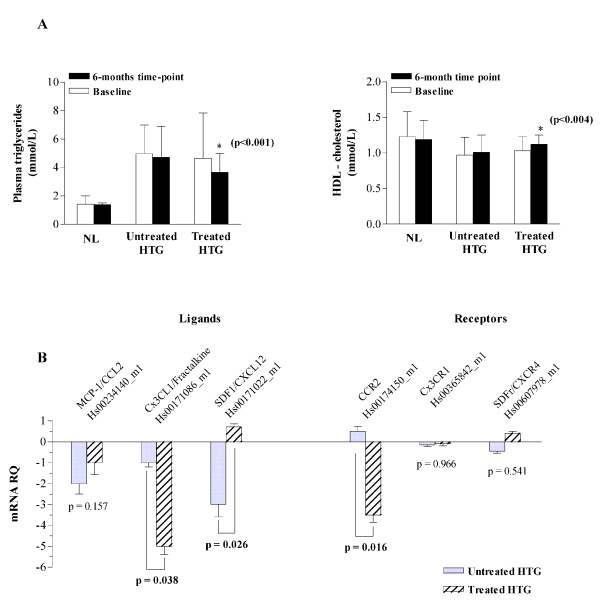
**(A) Effect of treatment with fenofibrate (160 mg/day) in HIV-infected patients with hypertriglyceridemia (treated HTG, n = 15) with respect to untreated normolipemics (NL, n = 43) and hypertriglyceridemics (untreated HTG, n = 24) subjects**. **(B) **The use of fenofibrate was related to significant decreases in the expression in circulating leukocytes of CX3CL1 and CCR2 genes and a significant increase in the expression of SDF-1.

Patients included in this cross-sectional study are participants of a previous study and the design has been already described [3]. Participants were selected among those on a PIs regimen and undetectable viral load during the previous 12 months. They were free of liver and renal disease. Forty-three patients were considered normotriglyceridemic and 39 hypertriglyceridemics (plasma values below or above the accepted threshold of 1.69 mmol/L). Fifteen of these hypertriglyceridemic patients were being treated with fenofibrate (160 mg/day) for at least six month, after having fulfilled the eligibility criteria (more than 6 months on stable HAART, more than 18 years of age, and a fasting triglyceride concentration >1.69 mmol/L). We collated pertinent HIV-related clinical and laboratory data as well as data on the course of plasma lipid profile from their clinical notes. Three mL of blood were collected into TEMPUS blood RNA tubes (ABI, Foster City, CA, USA) from which total RNA was isolated using the ABI PRISM 6100 (Applied Biosystems, Foster City, USA). TaqMan primers and probes were obtained from validated Assays-on-Demand products (Applied Biosystems) (Figure 1) to be used in a Micro Fluidic Card on the 7900HT Real Time PCR system. Micro Fluidic Cards were analysed with RQ documents and the RQ Manager Software for automated data analysis. Expression values for target genes were normalized to the concentration of a designated endogenous control (GAPDH). Gene expression values were calculated based on the comparative threshold cycle (Ct) method in which the samples belonging to the normotriglyceridemic group were designated as calibrators to each hypertriglyceridemic group and therefore the amount of transcripts were dimensionless numbers relative to the calibrator levels via the 2^-ΔΔCt ^method. We applied non-parametric tests when needed and logistic regression to test the association of gene expression with the use of fenofibrate.

## Results and Discussion

There were no significant differences among the three study groups for duration of HIV, age, body mass index and CD4+ T cell count. Although the challenge of adherence to drugs is substantial in routine HIV care, we found a good tolerance to fenofibrate, and no complaint was recorded. As expected, only in fenofibrate treated patients the decrease in fasting plasma triglyceride concentration was statistically significant as well as the increase in plasma HDL-cholesterol levels (Figure 1A). A reduction in plasma total cholesterol and MCP-1/CCL2 concentrations were also observed but it did not reach statistical significance.

With the rationale that the target for fenofibrate, PPARα, is expressed in leukocytes [7] we explored the mRNA expression of selected chemokine genes in whole blood. We found that with respect to untreated patients there was no change in the expression of chemokine receptors CX3CR1 and CXCR4 but the expression of CCR2 was significantly decreased in patients treated with fenofibrate (*U*-Mann-Whitney, p = 0.016; Figure 1B). Of note, CCR2 gene expression was positively correlated with CD4+ T cell count only in those patients that were undergoing fenofibrate treatment (Spearman, *ρ *= 0.7; p = 0.02). The expression of ligands for these receptors was clearly different. We did not find any significant effect of fenofibrate in the MCP-1/CCL2 gene expression. However, there was a significant decrease in the gene expression of the CX3CL1/Fractalkine in patients treated with fenofibrate with respect to untreated hypertriglyceridemic patients (*U*-Mann-Whitney, p = 0.038; Figure 1B). This is probably accompanied by decreased CX3CL1 plasma levels and a similar effect in the contiguous endothelial cells, which, in combination with the CCR2 decreased expression, could be beneficial with respect to the risk of atherosclerosis considering their well documented role in the local accumulation of monocytes [8,9]. However, fenofibrate had an intriguing effect in SDF-1 expression neutralizing the significant decrease in its expression observed in untreated hypertriglyceridemics (*U*-Mann-Whitney, p = 0.026; Figure 1B), presumably increasing SDF-1 values. SDF-1 plays a significant role in HIV infection because it is the natural ligand for CXCR4 which is used by HIV T-tropic strains to enter into the cells in advanced stages of the disease [10]. Additionally, although the interpretation of SDF-1 levels is difficult we have previously shown that the allelic variant SDF1-3'A appears to be protective against the development of carotid atherosclerosis [11].

In multivariate analysis, only fenofibrate treatment significantly contributed to explain CCR2 [*B *= 0.18 (0.003 to 0.09); P = 0.04] and SDF-1 [*B *= 4.6 (1.1 to 18.0); P = 0.03] expression levels in hypertriglyceridemic patients. This relationship, however, was not maintained in CX3CL1/Fractalkine expression [*B *= 0.23 (0.04 to 1.1); P = 0.07].

Therefore, our results show less lipid disturbances with the addition of fenofibrate in patients with HIV, which is accompanied by significant changes in the pattern of chemokine gene expression in circulating leukocytes. The data suggest that PPARα activators may be useful in the attempt to decrease the risk of atherosclerosis and may act as modulators of systemic inflammation and the associated vascular response. However, further studies in other type of populations without lipid abnormalities should be needed to confirm the fenofibrate effect or any lipid-lowering drug effect on systemic inflammation. Moreover, a note of caution should be added before the indication of these active drugs and further studies are needed to disregard potential deleterious effects on the HIV infection itself.

## List of abbreviations

Ct: threshold cycle; GAPDH: glyceraldehydes-3-phosphate dehydrogenase; HDL: high-density lipoprotein; MCP-1: monocyte chemotactic protein-1; PIs: protease inhibitors; PPARα: peroxisome proliferator-activated receptor α; RQ: relative quantification; SDF-1: stromal cell-derived factor-1.

## Competing interests

The authors declare that they have no competing interests.

## Authors' contributions

CA-V and JJ designed the study. CA-V, GA and RB-D analyzed the data. GA, RB-D, LF-S and AR collected data or performed various measurements for the study. CA-V wrote the first draft of the manuscript. JJ, JC and JMA contributed to the final version of the manuscript. GA, RB-D and AR helped in the data analysis and interpretation. JJ and JC supervised the laboratory determinations. All authors have read and approved the final manuscript.
